# Supplementary material for: Assessing parameter identifiability of a hemodynamics PDE model using spectral surrogates and dimension reduction
Source: PLoS Comput Biol. 2025 Oct 9;21(10):e1013553. doi: 10.1371/journal.pcbi.1013553 (PMC12520355; doi:10.1371/journal.pcbi.1013553)
Supplement: S1 File — Additional numerical results and profile-likelihood analyses for the five test data sets. (PDF) [file pcbi.1013553.s001.pdf]

Supplementary Material for: Assessing parameter identifiability of a hemodynamics model using spectral surrogates and dimension reduction

Mitchel J. Colebank[1,2*],

1 Department of Mathematics, University of South Carolina, Columbia, South Carolina, United States of America
2 Department of Biomedical Engineering, University of South Carolina, Columbia, South Carolina, United States of America

* mjcolebank@sc.edu

Signal variability

The one-dimensional (1D) hemodynamics model provides dynamics pressure ($p(x,t)$), flow ($q(x,t)$), and area ($A(x,t)$) along the length of a vessel. The two boundary condition types (Windkessel and structured tree) provide different types of boundary conditions, thus leading to slightly different signals. We also provide the standard deviation of each signal type for the Windkessel and structured tree model, shown in Fig A. The standard deviation is calculated from the training data for each boundary condition type.

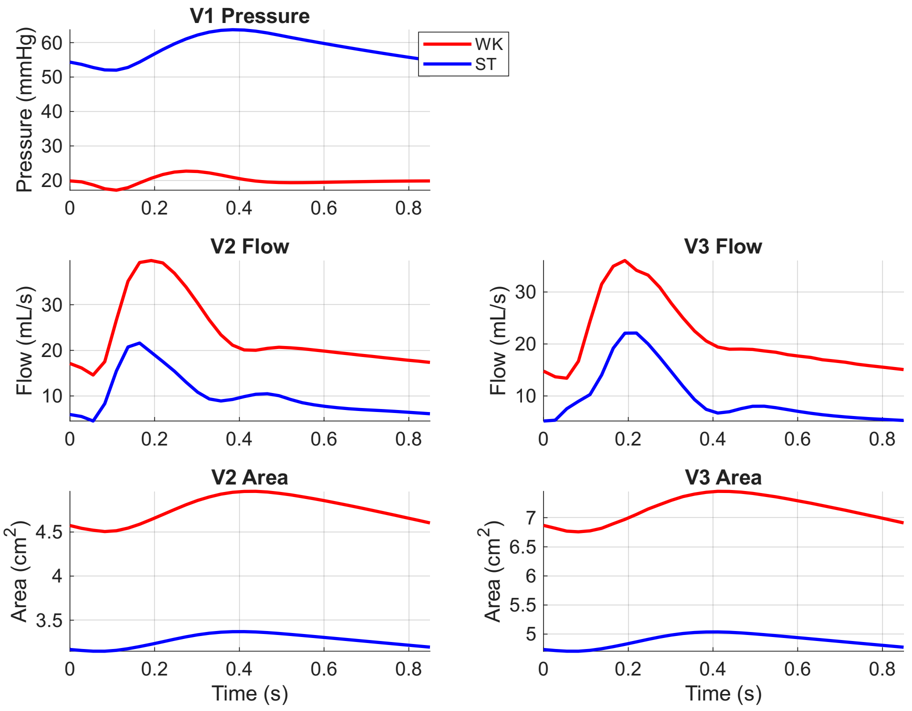

Fig A. Standard deviation calculated from the training data for the Windkessel (red) an structured tree (blue) boundary conditions and across the five signals used in the experimental designs in the main text.

Error metrics for dimension reduction and emulation

We build the polynomial chaos expansion (PCE) spectral surrogates using a reduced dimensional form of the original data. Principal component analysis (PCA) reduces the data to a mean vector and the most important directions as dictated from the data covariance matrix. We only retain a small subset of the orthonormal bases, thus there is inherent error in the signal reconstruction. There is additional error attributed to emulating the PCA scores themselves. We calculate the relative residual sum of squares (RRSS) for both errors via

$$RRSS_{em} \quad = \sum_{i=1}^{N} \left(\frac{\left(\boldsymbol{y}_i(t) - \mathcal{M}^{PCE}(t; \boldsymbol{\theta_i})\right)}{\max(\boldsymbol{y}_i(t))} \right) \tag{1}$$

$$RRSS_{pca} \quad = \sum_{i=1}^{N} \left(\frac{\left(\boldsymbol{y}_i(t) - \boldsymbol{y}_i^{pca}(t))\right)}{\max(\boldsymbol{y}_i(t))} \right) \tag{2}$$

We provide both of these metrics in Figs B and C below. In general, the emulation error is grater than the error induced by PCA, and the Windkessel model tends to have higher accuracy across both metrics compared to the structured tree. The structured tree pressure error related to emulation is one of the highest, followed by area. We also provide plots of the relative error between the emulator and signals corresponding to the experimental designs as a function of time in Figs D and E.

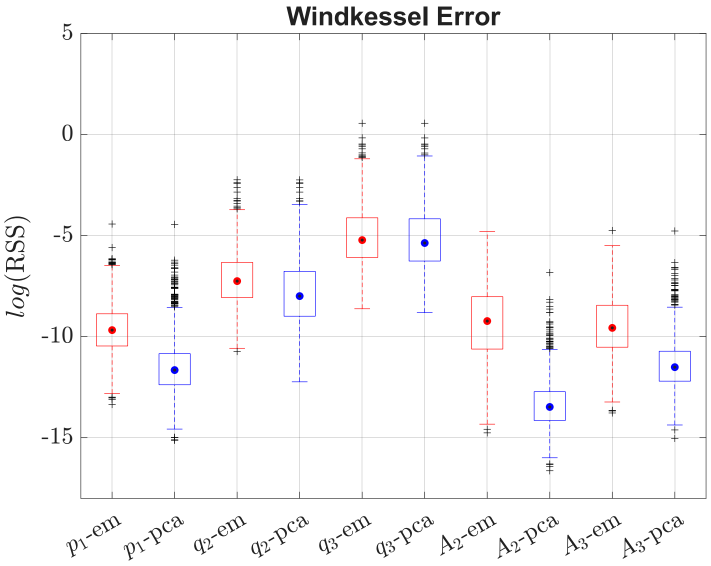

Fig B. Box-and-whisker plots comparing emulation error (subscript *em*, red boxes) and PCA dimension reduction error (subscript *pca*, blue boxes) for the Windkessel model. Errors are shown for the five signals used in the main text: pressure in the MPA (p_1), flow in the LPA and RPA (q_2 and q_3, respectively), and area in the LPA and RPA (A_2 and A_3, respectively).

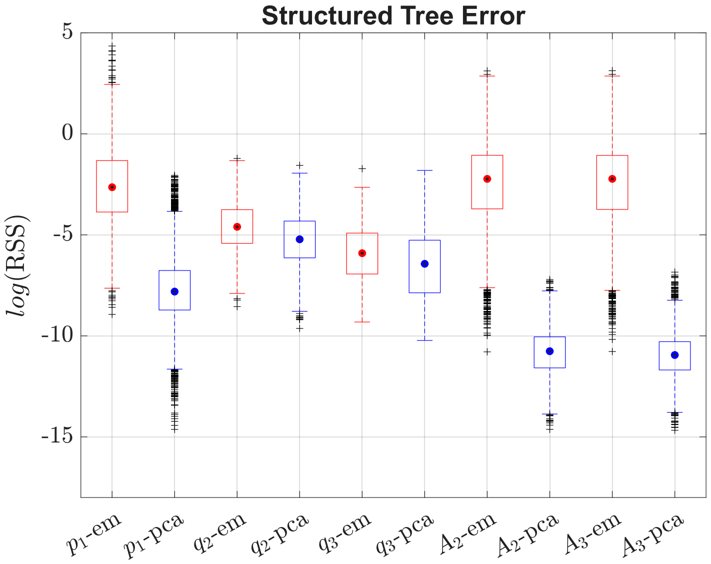

Fig C. Box-and-whisker plots comparing emulation error (subscript *em*, red boxes) and PCA dimension reduction error (subscript *pca*, blue boxes) for the structured tree model. Errors are shown for the five signals used in the main text: pressure in the MPA (p_1), flow in the LPA and RPA (q_2 and q_3, respectively), and area in the LPA and RPA (A_2 and A_3, respectively).

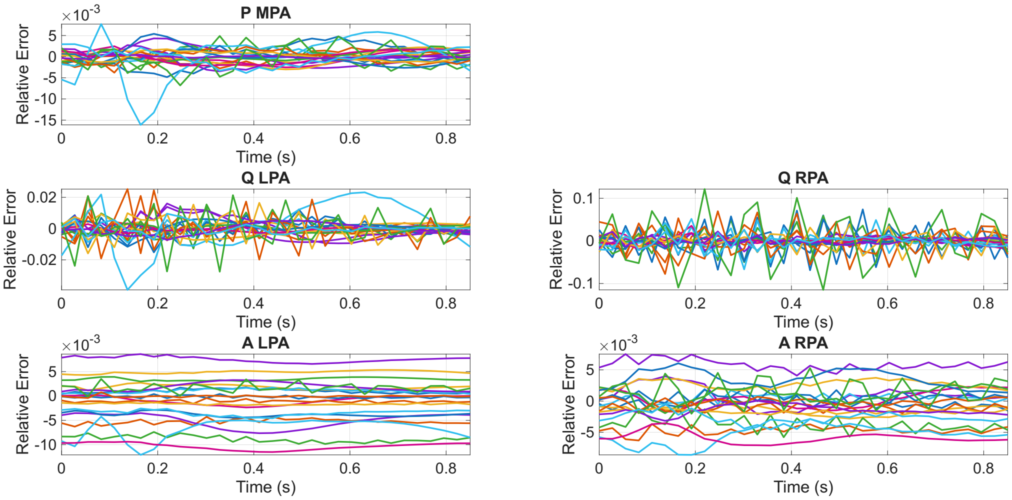

Fig D. Relative error (scaled by the maximum of the data) for the five output signals used in the experimental designs of the main text for the Windkessel boundary condition.

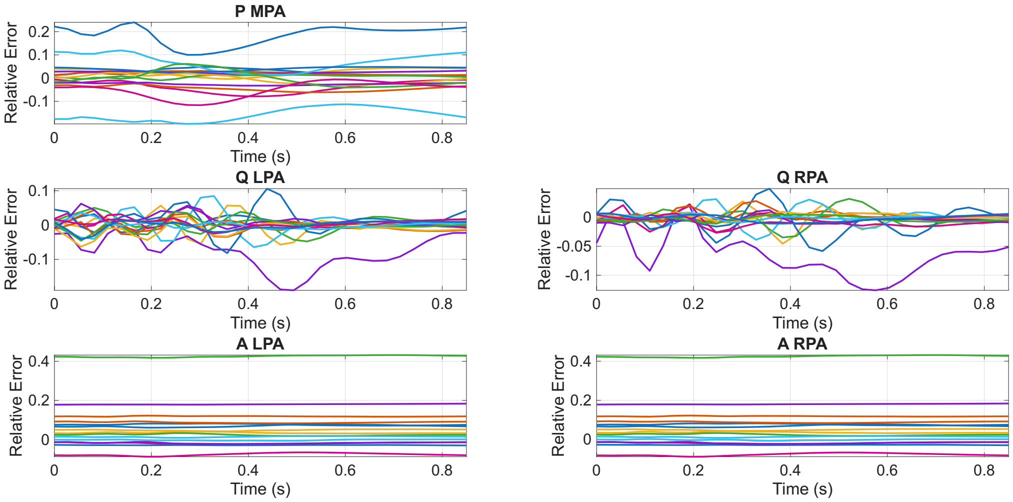

Fig E. Relative error (scaled by the maximum of the data) for the five output signals used in the experimental designs of the main text for the structured tree boundary condition.

Profile-likelihood results: Windkessel

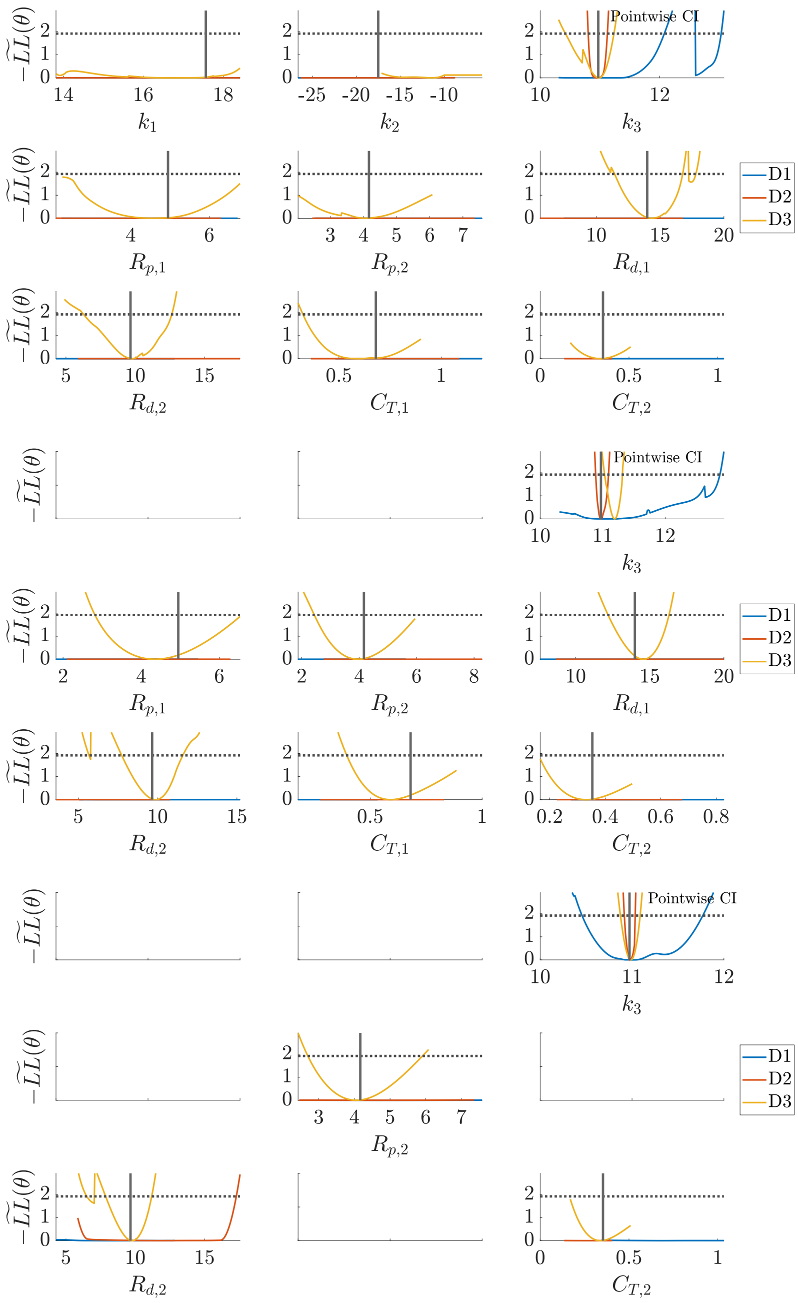

Fig F. Profile-likelihood results for test data set 2 using the PCA-PCE spectral surrogate. (a) Profile likelihood calculated using the three different experimental designs defined previously. The pointwise confidence intervals define whether parameters are considered identifiable. (b) A reduced parameter subset where k_1 and k_2 are not included in the profile-likelihood calculation. (c) A further reduced parameter set where k_1 and k_2 are fixed, as well as the LPA Windkessel parameters ($R_{p,1}$, $R_{d,1}$, and $C_{T,1}$). Blank plots represent parameters that are fixed.

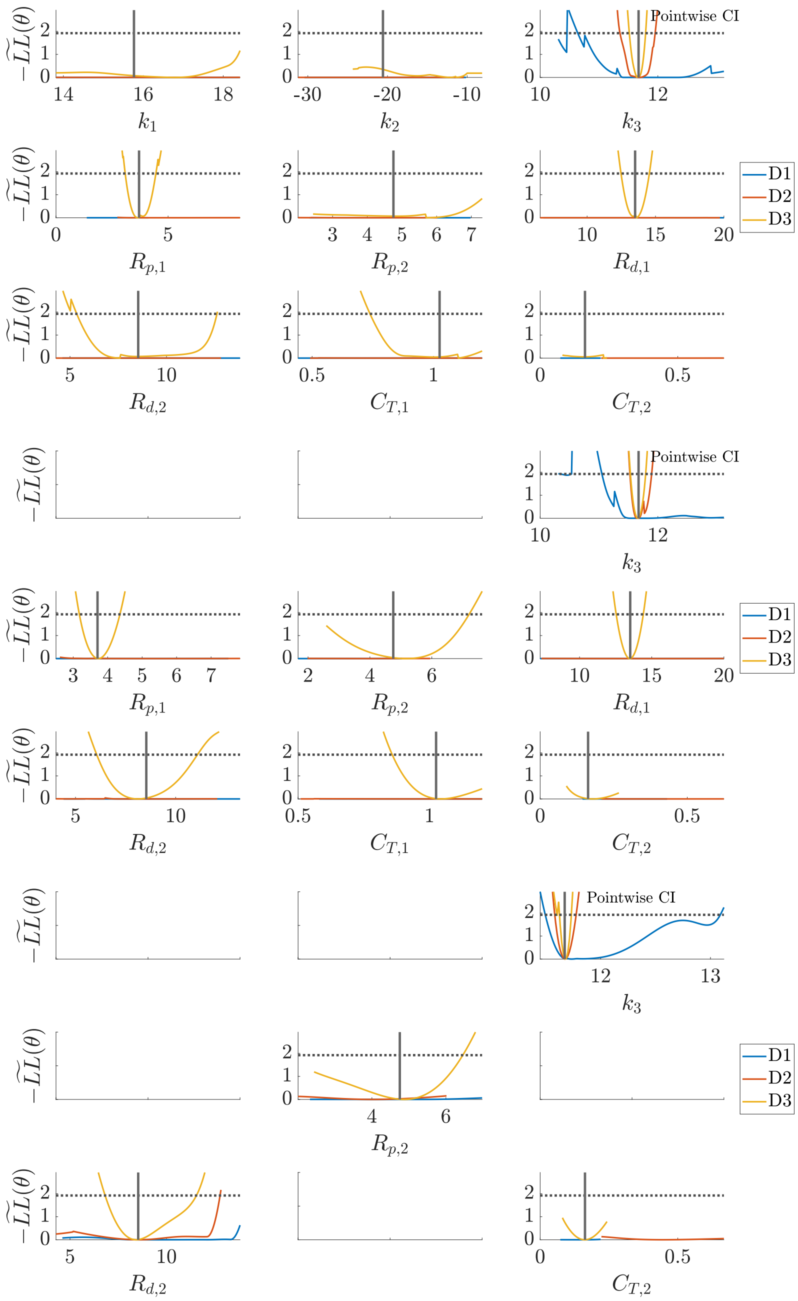

Fig G. Profile-likelihood results for test data set 3 using the PCA-PCE spectral surrogate. (a) Profile likelihood calculated using the three different experimental designs defined previously. The pointwise confidence intervals define whether parameters are considered identifiable. (b) A reduced parameter subset where k_1 and k_2 are not included in the profile-likelihood calculation. (c) A further reduced parameter set where k_1 and k_2 are fixed, as well as the LPA Windkessel parameters ($R_{p,1}$, $R_{d,1}$, and $C_{T,1}$). Blank plots represent parameters that are fixed.

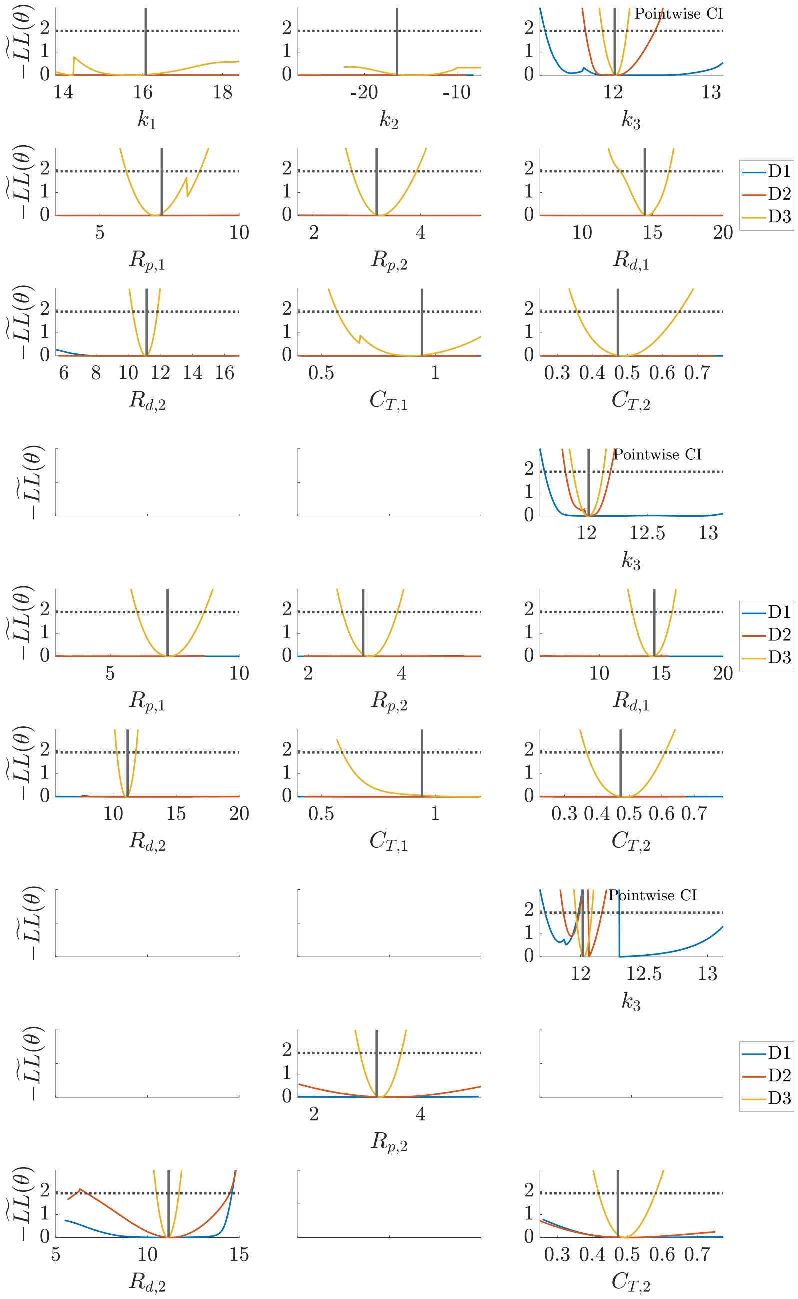

Fig H. Profile-likelihood results for test data set 4 using the PCA-PCE spectral surrogate. (a) Profile likelihood calculated using the three different experimental designs defined previously. The pointwise confidence intervals define whether parameters are considered identifiable. (b) A reduced parameter subset where k_1 and k_2 are not included in the profile-likelihood calculation. (c) A further reduced parameter set where k_1 and k_2 are fixed, as well as the LPA Windkessel parameters ($R_{p,1}$, $R_{d,1}$, and $C_{T,1}$). Blank plots represent parameters that are fixed.

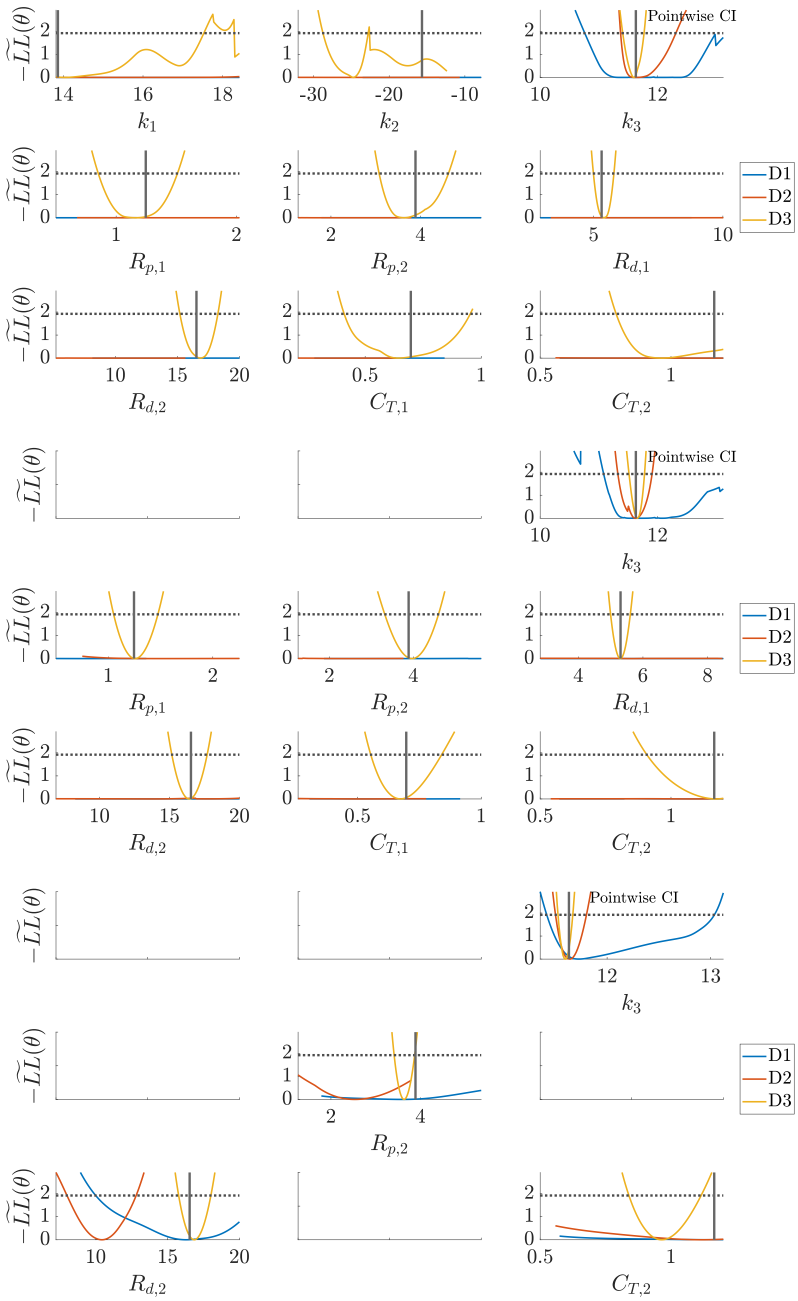

Fig I. Profile-likelihood results for test data set 5 using the PCA-PCE spectral surrogate. (a) Profile likelihood calculated using the three different experimental designs defined previously. The pointwise confidence intervals define whether parameters are considered identifiable. (b) A reduced parameter subset where k_1 and k_2 are not included in the profile-likelihood calculation. (c) A further reduced parameter set where k_1 and k_2 are fixed, as well as the LPA Windkessel parameters ($R_{p,1}$, $R_{d,1}$, and $C_{T,1}$). Blank plots represent parameters that are fixed.

Profile likelihood results: Structured tree

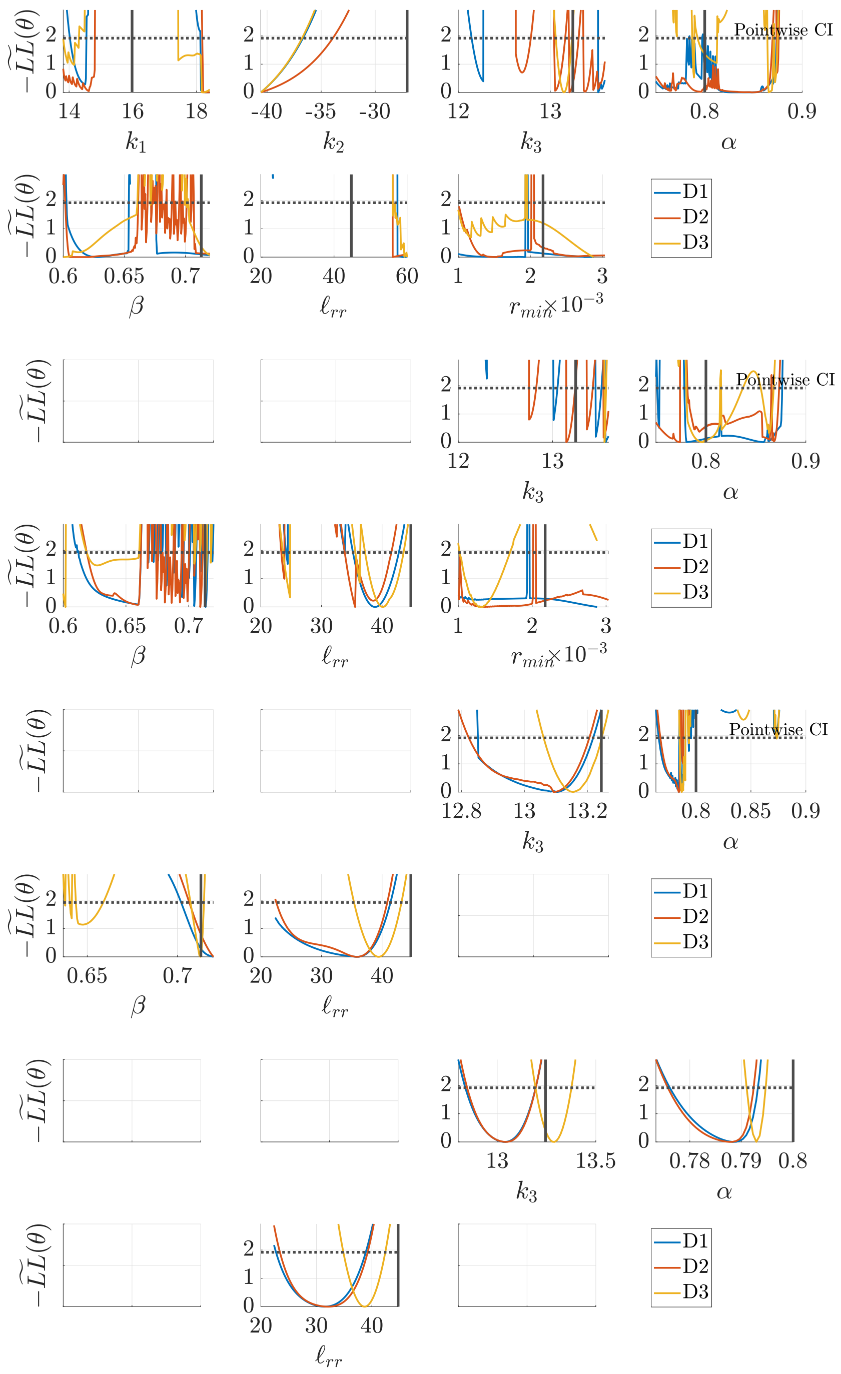

Fig J. Profile-likelihood results for test data set 2 using the PCA-PCE spectral surrogate. (a) Profile likelihood calculated using the three different experimental designs defined in previously. The pointwise confidence intervals define whether parameters are considered identifiable. (b) A reduced parameter subset where k_1 and k_2 are not included in the profile-likelihood calculation. (c) A further reduced parameter set where β is fixed. (d) Further reduced parameter set with r_{min} fixed. Blank plots represent parameters that are fixed.

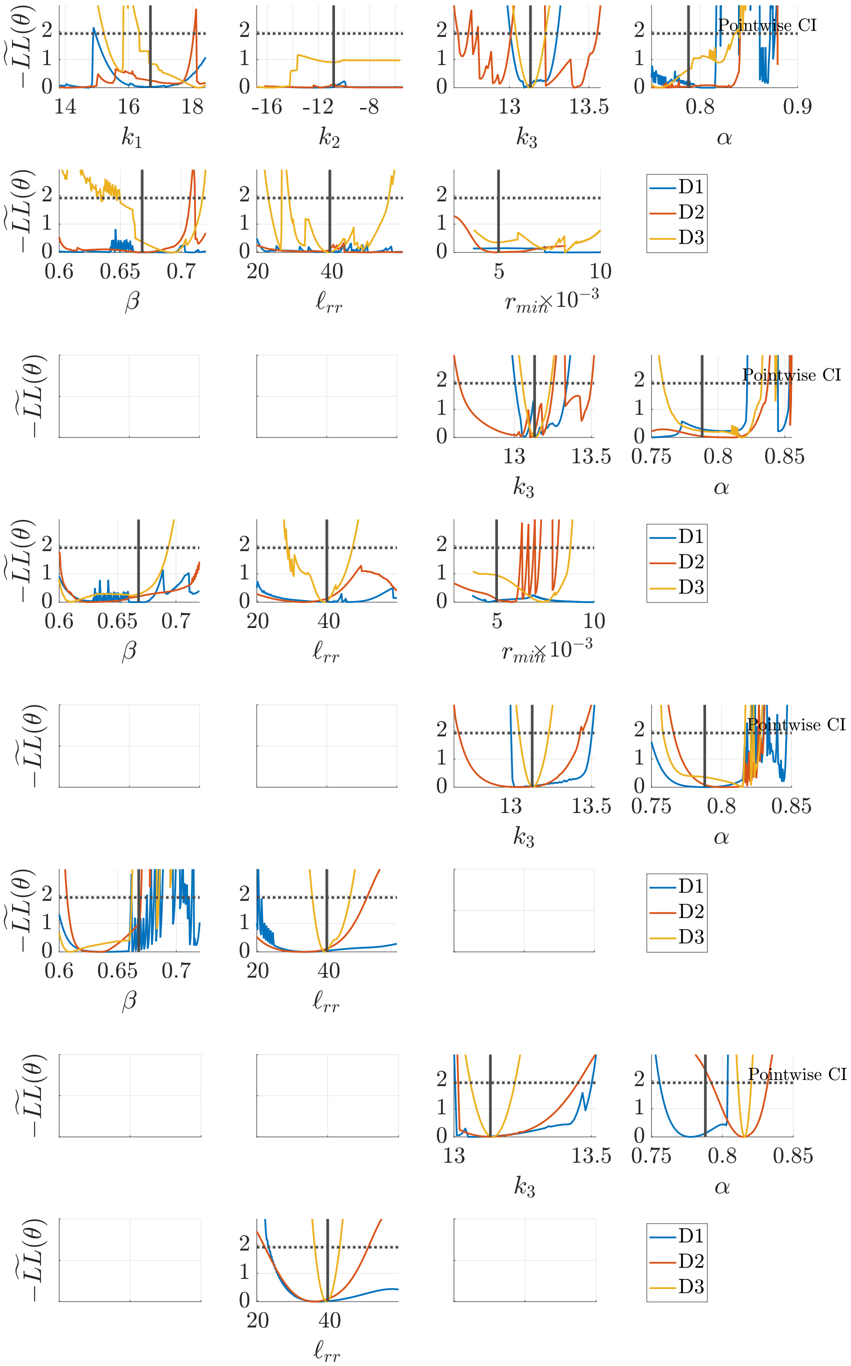

Fig K. Profile-likelihood results for test data set 3 using the PCA-PCE spectral surrogate. (a) Profile likelihood calculated using the three different experimental designs defined in previously. The pointwise confidence intervals define whether parameters are considered identifiable. (b) A reduced parameter subset where k_1 and k_2 are not included in the profile-likelihood calculation. (c) A further reduced parameter set where β is fixed. (d) Further reduced parameter set with r_{min} fixed. Blank plots represent parameters that are fixed.

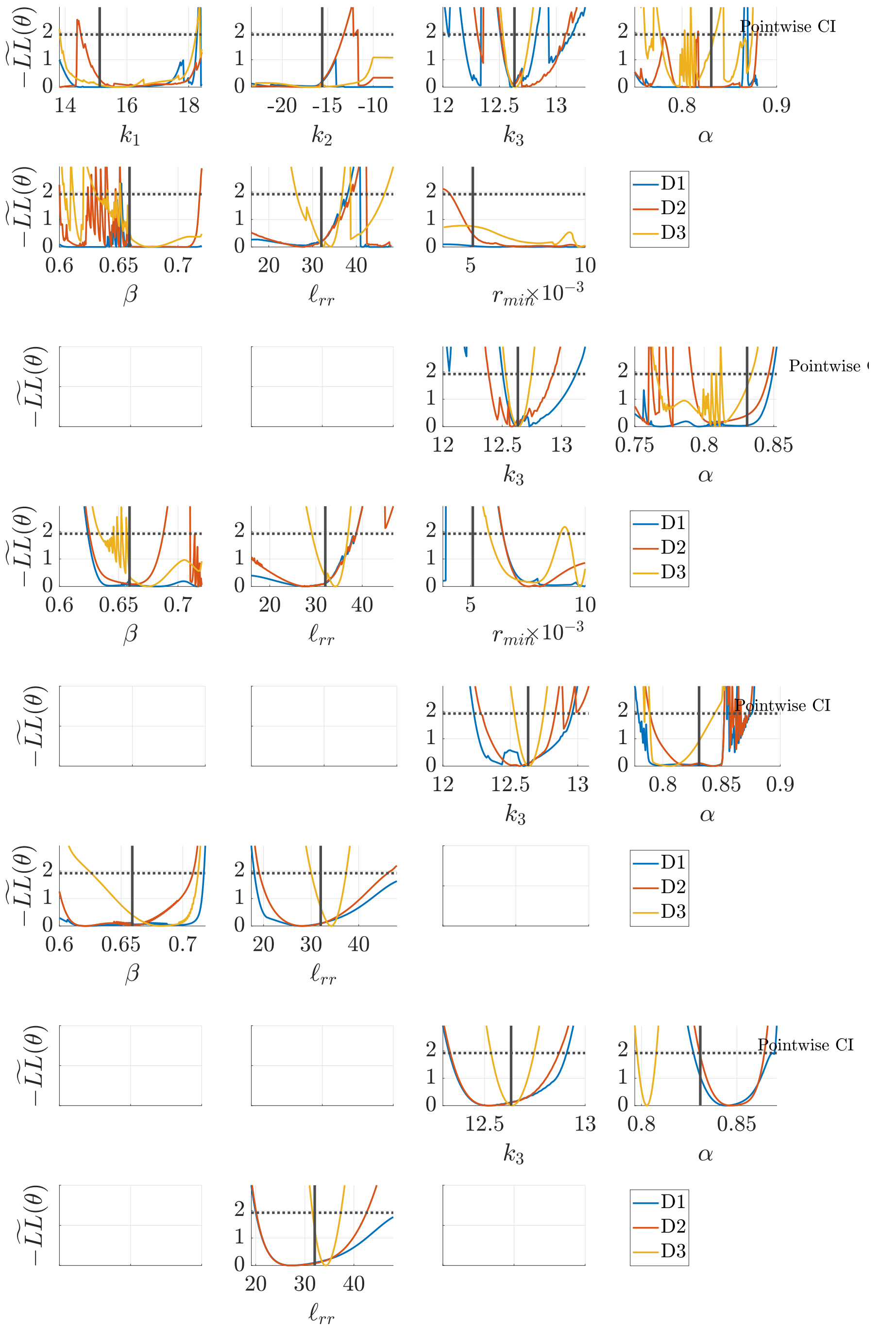

Fig L. Profile-likelihood results for test data set 4 using the PCA-PCE spectral surrogate. (a) Profile likelihood calculated using the three different experimental designs defined in previously. The pointwise confidence intervals define whether parameters are considered identifiable. (b) A reduced parameter subset where k_1 and k_2 are not included in the profile-likelihood calculation. (c) A further reduced parameter set where β is fixed. (d) Further reduced parameter set with r_{min} fixed. Blank plots represent parameters that are fixed.

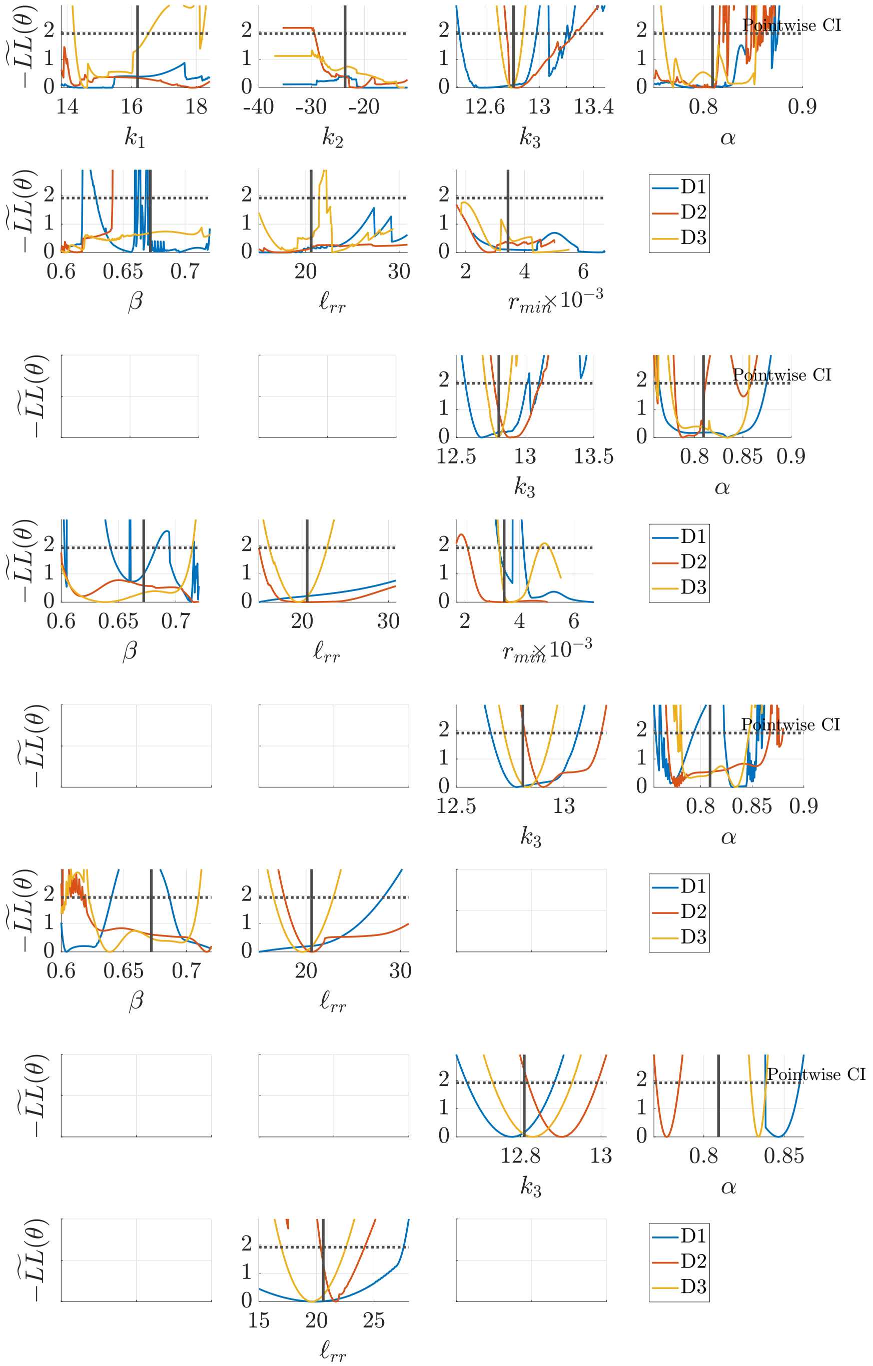

Fig M. Profile-likelihood results for test data set 5 using the PCA-PCE spectral surrogate. (a) Profile likelihood calculated using the three different experimental designs defined in previously. The pointwise confidence intervals define whether parameters are considered identifiable. (b) A reduced parameter subset where k_1 and k_2 are not included in the profile-likelihood calculation. (c) A further reduced parameter set where β is fixed. (d) Further reduced parameter set with r_{min} fixed. Blank plots represent parameters that are fixed.
