# Supplementary material for: Assessing parameter identifiability of a hemodynamics PDE model using spectral surrogates and dimension reduction
Source: PLoS Comput Biol. 2025 Oct 9;21(10):e1013553. doi: 10.1371/journal.pcbi.1013553 (PMC12520355; doi:10.1371/journal.pcbi.1013553)
Supplement: Attachment — Submitted filename: Paper_review.pdf [file pcbi.1013553.s002.pdf]

Comments for "Assessing parameter identifiability of a hemodynamics PDE model using spectral surrogates and dimension reduction", by Mitchel J. Colebank

June 2025

Overview

This work presents a detailed study regarding the surrogate modeling of hemodynamic PDEs. The novelty includes the study of using PCE-PCA emulators combined with reduced-order modeling for global sensitivity analysis as well as testing likelihood-based parameter identifiability. The impact of boundary conditions is also investigated through several UQ tasks. The analysis of the results are thorough, offering key implications to the studied pulmonary model.
We kindly suggest the author to revise the draft according to the following comments, carefully check for typos and inconsistencies, and provide more details if needed to improve the quality of this work.

Major comments

1. A concise introduction to the training/testing data of ROM and profile likelihood is missing. Any comments on the dataset size and split will be helpful.
2. During the simulation, how much the dynamic area of LPA and RPA can change? That may explain some parameter identifiability results. For example, the setting D3 leads to very different results than D1 and D2, while less variation is between D1 and D2.
3. Any comments on the numerical optimization method used for profile likelihood will be beneficial. In terms of algorithm, convergence, initial guess etc.
4. Comments on how the range of parameter perturbation for the method of profile likelihood will be useful. Why parameters like $R_{p,2}$, $R_{d,2}$, ℓ_{rr} are allowed to vary relatively more?
5. Page 8: the covariance matrix $\mathbf{\Sigma}$ provides non-dimensionalization and *models* the measurement noise. While no measurement noise is assumed in the current context, does this mean the discrepancy between emulator results and real data is modeled as a zero-mean Gaussian with diagonal co-variance matrix? If so, can this be related to the PCA setting?
6. The method of Profile likelihood can be expensive for high-dimensional and non-identifiable problems. The author may comment on how much speed-up can be gained via the surrogate modeling (e.g. results from Fig 11).
7. Page 16: *..., and may reflect issues with inferring the full parameter set prior to calculating the profile-likelihood confidence intervals.* Can the author provide more explanation on this remark?
8. Page 17: Figure 7 (c). Can the author comment on why a wider confidence interval (CI) is obtained for the design D2 (w.r.t parameter $R_{d,2}$ and $C_{T,2}$)? Comparing to D1, additional information is given in D2, hence its CI is supposed to become narrower. Additionally, a noticeable shift of the optimization result regarding the parameter $R_{p,2}$ is observed. Any potential explanation on this would be beneficial.

9. Page 18: Figure 8 (b). The likelihood profile results of D1 and D3 are very distinct, yet their corresponding output results are not as different as between D1 and D2. However, the profiling results of D1 and D2 are similar. Can the author provide further explanation on this?

10. Spatial information is averaged out in the current context. Additional comments on how to extend the future work into spatial-temporal framework will be helpful.

Minor comments

1. Page 4: *Windkessel models are a three-element electrical circuit....* The subject is plural.

2. Page 5: *...surrogate modeling technique that use standard polynomial types....* It should be *uses*.

3. Page 6: $c_q(\boldsymbol{\theta}$. The parentheses is unclosed.

4. Page 9: *..., as we the true date generating ...* There is a typo.

5. Page 11, Figure 3: Making the limits of y-axis uniform for 3(a) and 3(b) is recommended for easier comparison.

6. Page 14: *... components show that alpha, β, ...* latex typo here.

7. Page 16: *... and above the profile-likelihood confidence threshold in Figure 10.* There is a typo.
