# Supplementary material for: Assessing parameter identifiability of a hemodynamics PDE model using spectral surrogates and dimension reduction
Source: PLoS Comput Biol. 2025 Oct 9;21(10):e1013553. doi: 10.1371/journal.pcbi.1013553 (PMC12520355; doi:10.1371/journal.pcbi.1013553)
Supplement: Attachment — Submitted filename: Review_Round2.pdf [file pcbi.1013553.s004.pdf]

Comments for "Assessing parameter identifiability of a hemodynamics PDE model using spectral surrogates and dimension reduction", by Mitchel J. Colebank

Sept 2025

Major comments

1. **Original question**: Page 16: *..., and may reflect issues with inferring the full parameter set prior to calculating the profile-likelihood confidence intervals.* Can the author provide more explanation on this remark?

   Response to the author: In the tests associated to Figure 10, were the subsets obtained by fixing several parameters at their optimal values, i.e., the values produced by the initial *full parameter inference*? If so, unless considering convergence issue in numerical optimization, if the targets were the same for either inferring the full parameter set or the subsets, why the parameter α is considered *incorrectly* inferred within the D2 setting? What could be the reason behind this incorrectness? However, that might be simply due to an identifiability change w.r.t α after other parameters are frozen. This may also be implied by the drastic change of α's profile in Figure 10 (a,b,c,d).
