# Supplementary material for: Assessing parameter identifiability of a hemodynamics PDE model using spectral surrogates and dimension reduction
Source: PLoS Comput Biol. 2025 Oct 9;21(10):e1013553. doi: 10.1371/journal.pcbi.1013553 (PMC12520355; doi:10.1371/journal.pcbi.1013553)
Supplement: Attachment — Submitted filename: Reviewer_Reponse.docx [file pcbi.1013553.s003.docx]

Reviewer Rebuttal: “Assessing parameter identifiability of a hemodynamics PDE model using spectral surrogates and dimension reduction”, by Mitchel J. Colebank

I thank the reviewers for their constructive criticism and feedback on the submitted manuscript. Below is a point-by-point response to the reviewers’ comments (in **bold**). Changes to the manuscript are provided in ***red***.

**Reviewer 1**

**Overview**

This work presents a detailed study regarding the surrogate modeling of hemodynamic PDEs. The novelty includes the study of using PCE-PCA emulators combined with reduced-order modeling for global sensitivity analysis as well as testing likelihood-based parameter identifiability. The impact of boundary conditions is also investigated through several UQ tasks. The analysis of the results are thorough, offering key implications to the studied pulmonary model.

We kindly suggest the author revise the draft according to the following comments, carefully check for typos and inconsistencies, and provide more details if needed to improve the quality of this work.

**Major comments**

1. A concise introduction to the training/testing data of ROM and profile likelihood is missing. Any comments on the dataset size and split will be helpful.

- **We agree that this is missing from the present manuscript. We have updated the text on page 10 in the Results section to include this information regarding the testing/training split. For the profile-likelihood, the same surrogates are used, but we focus on five of the one hundred testing data sets. The text describing the training/testing data split on page 10 now reads:**

“*We generate 2500 samples to train the Windkessel boundary condition model, of which 2470 successfully run through the simulator without numerical convergence issues. We use a relatively larger number of samples for the structured tree model, given its larger pressure variance (see Supplementary Material). We generate 3000 samples for structured tree emulation, all of which run successfully. For testing error and later profile-likelihood calculation, we generate 50 test datasets for each boundary condition type.”*

2. During the simulation, how much the dynamic area of LPA and RPA can change? That may explain some parameter identifiability results. For example, the setting D3 leads to very different results than D1 and D2, while less variation is between D1 and D2.

**- We have provided a new Fig 3 in the main text showing seven realizations from the training data for the Windkessel and structured tree boundary conditions. In addition, we provide the standard deviations, calculated from the entire training dataset, in Fig 1 of the supplementary material. The area dynamics for each vessel are tightly coupled to the pressure dynamics, given the pressure-area relationship described in eq. (4) of the main text. Since pressure dynamics are only slightly different in this relatively small network, area dynamics are roughly the same except for area magnitude. The figure below shows the difference between the parent and daughter vessels in terms of pressure, area, and relative area (i.e., strain). This shows that the differences in pressure and area between the branches are distinct, but the strain pattern (which is corrected for area magnitude) is similar to pressure. Thus, there is coupling between pressure and area via eq. (4) which likely explains why designs** $\boldsymbol{D}\boldsymbol{2}$ **is only minimally informative given that pressure and area are tightly coupled.**

**Changes in pressure (top), area (middle), and strain (bottom) between MPA and LPA vessels or MPA and RPA vessels.**

**We have expanded on this finding in the Discussion section on page 28:**

*“We see that, in general, D2 is only minimally informative over D1 in terms of profile-likelihood results. We attribute this finding to the strong coupling between pressure and area as given by eq. (4). Pressure and area signals are nearly identical in shape in all three branches, as shown in Fig 3, though the area magnitudes are unique for the three branches. The sensitivity results in Figs 6 and 7 also show that pressure and area have similar dynamic sensitivities and top parameters influencing their dynamics. This is in contrast to flow, used in D3, which is distinct from pressure and area, and coupled through more complex mechanisms in the PDE equations themselves.”*

3. Any comments on the numerical optimization method used for profile likelihood will be beneficial. In terms of algorithm, convergence, initial guess etc.

**- We agree that this information will be useful for replication purposes. We have updated these details in the Methods section on page 10:**

*“We calculate univariate profile-likelihood confidence intervals by minimizing eq. (28) using the Levenberg–Marquardt algorithm in MATLAB's* ***lsqnonlin.m*** *package. We use a finite difference step size of* ${10}^{-4}$ *and a maximum of 300 iterations for each profiled parameter value.”*

4. Comments on how the range of parameter perturbation for the method of profile likelihood will be useful. Why parameters like Rp,2, Rd,2, ℓrr are allowed to vary relatively more?

**- We thank the reviewer for noticing this, as the details about parameter bounds were not detailed. For each numerical experiment, we first infer all of the model parameters using the data corresponding to the experimental design. Then, from this optimal set, we assign the upper and lower bounds for the profiled parameters to be** $\boldsymbol{\pm50\%}$ **of the inferred parameter value. This is why certain parameters and instances (e.g.,** $\boldsymbol{D}\boldsymbol{1, D}\boldsymbol{2, D}\boldsymbol{3}$ **for different test data) have different ranges for where parameters are profiled.**

**We have included this information in the Methods section on page 10:**

*“The profile-range for each parameter is calculated as* $\pm50\%$ *of the optimal parameter estimate and thus varies with each experimental design and test dataset.”*

5. Page 8: the covariance matrix Σ provides non-dimensionalization and models the measurement noise. While no measurement noise is assumed in the current context, does this mean the discrepancy between emulator results and real data is modeled as a zero-mean Gaussian with diagonal co-variance matrix? If so, can this be related to the PCA setting?

**- The reviewer is correct: we use simulator data without any added noise, thus the error obtained from the optimization and profile-likelihood routine is strictly related to surrogate/PCA error and is implicitly assumed to be a zero-mean, Gaussian with no cross-covariance terms. We scaled the likelihood by the mean of the data primarily to ensure that units and magnitudes do not weigh certain signals more than others (e.g., pressures in the range 8-40 mmHg, areas in the range 2-8 cm^2^ and flows in the range 0-400 mL/s).**

**The relationship between the error induced by dimensionality reduction versus emulation error is interesting to consider, given that the emulation is inherently based on a reduced dimensionality dataset. The figure below shows a comparison between two different metrics: the relative residual sum of squares (**$\boldsymbol{RRSS}$**) attributed to emulation and PCA. These are**

$$RRSS_{em}=\sum_{i=1}^{N} \left( \frac{\left( y_{i}\left( t \right)-\mathcal{M}^{PCE}\left( t;\boldsymbol{\theta}_{i} \right) \right)}{\max\left( y_{i}\left( t \right) \right)} \right)^{2}$$

$$RRSS_{pca}=\sum_{i=1}^{N} \left( \frac{\left( y_{i}\left( t \right)-y_{i}^{PCA}\left( t \right) \right)}{\max\left( y_{i}\left( t \right) \right)} \right)^{2}$$

**which quantify the relative error between the simulator signals (**$\boldsymbol{y}_{\boldsymbol{i}}\left( \boldsymbol{t} \right)$**) and either the PCA-PCE surrogate (**$\mathcal{M}^{PCE}\left( t;\theta_{i} \right)$**) or the reconstructed signals using only 5 principal components (**$y_{i}^{PCA}\left( t \right)$**). We provide results on a log-scale to make comparisons easier. As shown below, the error induced by PCA is consistently smaller than that attributed to emulation, consistent with intuition. However, we do see that signals with smaller PCA reconstruction error (the** $\boldsymbol{A}_{\boldsymbol{2}}$ **and** $\boldsymbol{A}_{\boldsymbol{3}}$ **predictions used in** $\boldsymbol{D}_{\boldsymbol{2}}$ **in the main text) have lower emulation errors as well. However, we can conclude that the structured tree model is harder to (i) reconstruct by PCA and (ii) to emulate than the Windkessel model. Taking into account both errors *a priori* is an additional avenue that we will consider moving forward.**

**We have added these results to the Supplementary Material on pages 1-4. In addition, we added the following text to the Results section on pages 10-11:**

*“We also computed the relative square error (similar to eq.(31)) attributed to the PCA reconstruction and the emulation of PCA reduced signals. These results, located in the Supplementary Material, show that the Windkessel model is substantially easier to approximate by PCA than the structured tree. In addition, the error induced by dimension reduction is similar in magnitude to the error from emulation, whereas pressure and area signals tend to have much higher emulation errors relative to reconstruction errors by PCA.”*

**As well as new text in the Limitations section on page 29:**

*“There is inherent approximation error induced by dimension reduction, which affects emulation accuracy on the true time-series signals. Thus, future work embedding this dimension reduction discrepancy into the solution to an inverse problem should be investigated [27].”*

**Log residual sum of squares (RSS) for the Windkessel model attributed to emulation (red) versus dimension reduction (blue)**

**Log residual sum of squares (RSS) for the structured tree model attributed to emulation (red) versus dimension reduction (blue)**

6. The method of Profile likelihood can be expensive for high-dimensional and non-identifiable problems. The author may comment on how much speed-up can be gained via the surrogate modeling (e.g. results from Fig 11).

**- We agree that this information is important for emphasizing the novelty of this work. The Windkessel model takes approximately 3 seconds per forward solve. The structured tree model takes on average 6 seconds per forward solve, though this can reach upwards of 20-30 seconds for certain parameter combinations. The Windkessel PCE evaluation takes at most 0.1 seconds while the structured tree PCE evaluation takes roughly 0.05 seconds. When calculating the profile-likelihood for the Windkessel or structured tree models, we found that a single optimization routine in the profile-likelihood procedure with non-identifiable parameters may take upwards of 300 to 2300 PCE evaluations, a maximum of 230 or 115 seconds for the Windkessel or structured tree PCEs. This would require 115-230 minutes with the PDE models themselves. Thus, the use of PCEs reduce the time for a full analysis using profile-likelihood from the order of days to weeks (even with parallel computing power) to the order of hours, and makes it feasible to test different experimental designs and the effects of parameter fixing. Moreover, the training data for PCEs can be produced with massive parallelization and simulations can be stored such that different surrogates and experimental designs can be examined after an initial set of “offline” PDE solves.**

**We have updated the text in the Discussion section on page 26 to reflect this significant time reduction:**

*“Computing the solution to the PDEs using Windkessel or structured tree boundary conditions take roughly 3 and 6 seconds, respectively. Non-identifiable parameter sets may require 300 to more than 2000 function evaluations per optimization, and this must be repeated upwards of 100-200 times for each profiled parameter. This is on the order of days or weeks to provide the full profile-likelihood for the full parameter set with a single experimental design. In contrast, generated training data for PCEs can be done in parallel, expediting this process substantially, and the evaluation of the PCEs themselves take 0.1 and 0.05 seconds for the Windkessel and structured tree surrogates, respectively.”*

**We also added the following to the conclusion section on page 29:**

*“By using PCEs, we drastically reduce the computation time required for the profile-likelihood from the order of days to the order of hours.”*

7. Page 16: “..., and may reflect issues with inferring the full parameter set prior to calculating the profile likelihood confidence intervals.” Can the author provide more explanation on this remark?

**- In our approach, we begin all profile-likelihood experiments by first trying to infer the entire parameter set, regardless of whether the entire set is identifiable, rather than fixing non-identifiable parameters at their true value. We do this so that the results are consistent with future studies using real data, as the full parameter set will need to be adjusted to the data in the absence of a “true” parameter value. Then, we profile each parameter around the optimal parameter set. Since we start by inferring all the parameters, there is a chance that the parameter value we are profiling around may be wrong, and hence the minimum of the confidence interval may not coincide with the true data-generating parameter set. Our results would provide ideal profile-likelihoods if we were to fix the non-identifiable parameters at the data-generating value; however, this limits extension of these methods to real-data, as we do not know the data-generating parameters.**

**We have elaborated more on this in the Methods section on page 10:**

*“Our profile-likelihood routine is carried out as follows. We start by inferring the full set of parameters, including those that are considered non-identifiable, to replicate the procedure that would be taken with actual measurements. We analyze the results from the initial univariate profile-likelihood across all parameters in combination with global sensitivity results to determine whether there are non-identifiable parameters that may be fixed. We subsequently analyze the univariate profile-likelihoods for smaller parameter subsets. This is repeated across the three experimental designs* $D1,D2$*, and* $D3$*. This reflects a more realistic scenario than fixing parameters at their true value prior to inference, as the true data generating parameters are always unknown.”*

**We also updated the text in the Results section explaining the above comment on page 20:**

*“The parameter* $\alpha$ *is incorrectly identified using* $D2$ *during the initial inference procedure, hence the univariate profile-likelihood confidence bounds are located far away from the results using* $D1$ *and* $D3$*.”*

8. Page 17: Figure 7 (c). Can the author comment on why a wider confidence interval (CI) is obtained for the design D2 (w.r.t parameter Rd,2 and CT ,2)? Comparing to D1, additional information is given in D2, hence its CI is supposed to become narrower. Additionally, a noticeable shift of the optimization result regarding the parameter Rp,2 is observed. Any potential explanation on this would be beneficial.

**- We agree that this finding is somewhat non-intuitive. When switching from** $\boldsymbol{D}\boldsymbol{1}$ **to** $\boldsymbol{D}\boldsymbol{2}$**, there is an increase in available data for inference. However, this also means that the likelihood function has changed to include area data, and profile-likelihood based confidence bounds are now based on a weighted combination of pressure and area (or pressure and flow in** $\boldsymbol{D}\boldsymbol{3}$**). This makes it difficult to conclude that more data will directly reduce profile-likelihood confidence intervals, since they should be conditioned on the likelihood values which change with different experimental designs.**

**To illustrate this, we have included the three figures below for MPA pressure, LPA area, and RPA area predictions corresponding to the results in Fig 8(c). This shows that a wider confidence interval does not necessarily imply a smaller uncertainty in the output space (especially with respect to area predictions). This is likely a consequence of changing the likelihood function with the experimental designs. We view this result as an interesting next step, where more attention may need to be focused towards devising robust likelihood functions to use in the profile-likelihood when experimental designs vary.**

**We have expanded on this in the Discussion section on page 28:**

*“In a similar light, we also see that increasing the available data is not guaranteed to decrease the parameter confidence bounds, as shown in Fig 8(c). Though design* $D2$ *provides area data, the confidence bounds for* $R_{p,2}$*,* $R_{d,2}$ *, and* $C_{T,2}$ *appear to widen relative to* $D1$*. We attribute this finding with the necessary changes in the likelihood function used in eq. (28). Including additional non-dimensionalized data in the likelihood shifts the calculation of the confidence intervals from pressure-only to a weighted combination of pressure and area (or flow) data. Hence, the profile-likelihood widths may need to be compared with this in mind.”*

**We have also added these findings in our Limitations section on page 29:**

*“There is also a need for more robust methods for updating the likelihood function based on new experimental data. This includes more statistically informed methods for scaling multiple entries in the likelihood function.”*

**Evaluations along the profile-likelihood for the three different designs for pressure in the MPA (top), area in the LPA (middle), and area in the RPA (bottom) for the four parameter subset presented in Fig 8(c).**

9. Page 18: Figure 8 (b). The likelihood profile results of D1 and D3 are very distinct, yet their corresponding output results are not as different as between D1 and D2. However, the profiling results of D1 and D2 are similar. Can the author provide further explanation on this?

**- The author is correct that the confidence intervals in parameter space and corresponding simulations in output space do not coincide. This is related in part to the concept of parameter influence versus identifiability. We show in Fig 5(a-c) that** $\boldsymbol{k}_{\boldsymbol{1}}$ **is not influential on pressure, area, or flow, with the exception of the third principal component of flow. Since this parameter is non-influential, it has little to no effect on the model predictions, hence the profiles in Fig 9 (a) are flat except for** $\boldsymbol{D}\boldsymbol{3}$**, which uses flow data (where it is marginally influential). In contrast, the parameters** $\boldsymbol{k}_{\boldsymbol{3}}$ **and** $\boldsymbol{R}_{\boldsymbol{d,1}}$ **are some of the most influential, and small changes in their values cause large deviations in the simulated outputs. The reason that** $\boldsymbol{R}_{\boldsymbol{d,1}}$ **shows no noticeable changes in output space in** $\boldsymbol{D}\boldsymbol{1}$ **and** $\boldsymbol{D}\boldsymbol{2}$ **is because it is non-identifiable with these designs, and can be compensated by other parameters in the system (e.g.,** $\boldsymbol{R}_{\boldsymbol{p,1}}$ **or** $\boldsymbol{R}_{\boldsymbol{d,2}}$**). Finally,** $\boldsymbol{k}_{\boldsymbol{3}}$ **is both highly influential (shown in Fig 5) and it is uniquely determined by pressure data (it controls the stiffness of the blood vessels). Hence this parameter always obtains narrower confidence bounds and it has large effects on the model output of pressure. These three examples provide (i) one non-influential and hence non-identifiable parameter in** $\boldsymbol{k}_{\boldsymbol{1}}$**, (ii) one influential, but often non-identifiable parameter in** $\boldsymbol{R}_{\boldsymbol{d,1}}$**, and (iii) an influential and identifiable parameter in** $\boldsymbol{k}_{\boldsymbol{3}}$**.**

**To emphasize this, we have added the following text in the Discussion section on page 27:**

*“Evaluations of the model along the univariate profile-likelihood, shown in Fig 9, provide a distinction between non-influential and non-identifiable parameters. First, the low sensitivity of* $k_{1}$ *provided in Fig 5 implies this parameter is non-influential. Thus, profiling this parameter has little to no effect on pressure (or area, with minimal effects on flow) and thus the output signals lay on top of each other. This corresponds to non-identifiability driven by the small influence of the parameter. Area and flow predictions (not shown) also overlay for designs* $D2$ *and* $D3$*, respectively, reflecting the effects of non-identifiable parameters on output signals. The sensitivity results for* $R_{d,1}$ *show that it is highly influential, and thus affects pressure, area, and flow predictions. However,* $R_{d,1}$ *interacts with other parameters of the system (e.g.,* $R_{p.1}$ *and* $R_{d,2}$*), especially in its effects on pressure and area. This is why the univariate profile-likelihood for* $R_{d,1}$ *indicates non-identifiability using designs* $D1$ *and* $D2$*. Once flow data is included in* $D3$*,* $R_{d,1}$*becomes identifiable. This is due in part to the importance of resistance on controlling flow distribution between the two branches [17,35]. Finally, sensitivity results show that* $k_{3}$ *is influential on pressure, flow, and area. Previous studies have also found that* $k_{3}$ *is pivotal in controlling the time-series dynamics of pressure [19,20], hence it is uniquely identifiable across all three designs given that pressure data is consistently used.”*

10. Spatial information is averaged out in the current context. Additional comments on how to extend the future work into spatial-temporal framework will be helpful.

**- The PDE model considered in this work does include a spatial component, though we fix the spatial location (the midpoint in each vessel) for all analyses. We agree that methods that address both high spatial and temporal resolution need further development. We employ PCA, though proper orthogonal decomposition (POD) is often used for engineering applications where spatial domains are more expansive. We acknowledge that future work should address explicit spatial variability, though we believe our approach can be applied in a similar manner.**

**We have thus updated these details in the Limitations section on page 29:**

*“Moving forward, we will apply similar methods to PDEs with higher spatial-dimensionality [38].”*

**Minor comments**

1. Page 4: Windkessel models are a three-element electrical circuit.... The subject is plural.

**- We have updated the text to read***: “Windkessel models are three-element electrical circuit analogs”*

2. Page 5: ...surrogate modeling technique that use standard polynomial types.... It should be uses.

**- We have updated the text.**

3. Page 6: cq(θ. The parentheses is unclosed.

**- We have updated the text.**

4. Page 9: ..., as we the true date generating ... There is a typo.

**- We have updated the text.**

5. Page 11, Figure 3: Making the limits of y-axis uniform for 3(a) and 3(b) is recommended for easier comparison.

**- We have updated Fig 4 (previously Fig 3) to include the same y-limits.**

6. Page 14: ... components show that alpha, β, .... latex typo here.

- **We have addressed this typo.**

7. Page 16: ... and above the profile-likelihood confidence threshold in Figure 10. There is a typo.

**- We intend for this text to imply that we are plotting model solutions that are either below the confidence level (i.e., below the profile-likelihood limits) or above the confidence level. We have now updated the text so that the sentence reads:** “Similar to Fig 10, *we provide output signals that are within the univariate profile-likelihood confidence threshold, as well as signals that are beyond the confidence limits, in Fig 12***.”**

**Reviewer #2:**

The submission "Assessing parameter identifiability of a hemodynamics PDE model using spectral surrogates and dimension reduction" addresses the important question of parameter estimation, identifiability and sensitivity analysis for a PDE model of blood flow. While the work seems to be of interest and technically correct, the current presentation of the work speaks to a narrow audience (e.g. blood flow modellers) and with some additional care the work could be appreciated by a much broader audience. My comments are intended to help broaden out the appeal of the work since I expect that the findings and the methodology will be of broad interest beyond those computational scientists working in blood flow modelling. I have some comments:

1. The introduction rightly points out that many methods and results for calibration, identifiability analysis and experimental design are known. In addition, working with profile likelihood can be powerful to explore questions of parameter identifiability, yet few studies have attempted this tool for PDE models (i.e. much more has been done for ODE models). References 14-16 are examples where PDE models are studied using PL. The authors could mention https://doi.org/10.1098/rsif.2020.0055 which pre-dates all of the references given in the introduction and the authors certainly use PL to consider identifiability.

**- We agree that we have missed several earlier articles that discuss profile-likelihood based identifiability in the context of PDE models. The aforementioned reference and one earlier reference by Boiger et al. (doi: 10.1088/0266-5611/32/12/125009) have now been included in the Discussion section. The text on page 26 now reads:**

*“While considered the gold standard for practical identifiability [12, 13, 35], these methods are computationally expensive and rarely used with PDE models [14, 16]. Some notable examples of this include the study by Boiger et al. [18], who considered an integration-based approach for the profile likelihood calculation for inverse problems with PDE constraints. The authors considered a Lagrangian formulation from which they could leverage adjoints for obtaining the profile-likelihoods. The study by Simpson et al. [17] compared the profile-likelihood with Bayesian inference in the context of spatio-temporal cell movement. The authors found that parameters of a reaction-diffusion like model were practically non-identifiable when diffusion parameters were cell population specific.”*

2. Throughout the MS (both in the intro and methods), Colebank refers to PL as being for one parameter at a time. This is not incorrect, but it is also worth noting that PL can be constructed for pairs and triples of parameters so these statements can be slightly adjusted to give a proper definition.

**- We agree that providing information about pairwise and multivariate profile-likelihood is worth mentioning. Throughout the text we have tried to emphasize that our methods focus on “univariate profile-likelihood.” The Introduction section on page 2 now reads:**

*“The profile-likelihood is a powerful identifiability method, which assesses which*

*parameters are identifiable by constructing confidence intervals [2, 12, 13]. Typically*

*these confidence intervals are calculated for one parameter at a time, though it is*

*feasible to profile multivariate parameter combinations as well.”*

**We also added the following text to the Methods section on page 8:**

*“The profile-likelihood is a frequentist statistical method that explicitly calculates*

*parameter confidence intervals by “profiling” parameters, i.e. by fixing some at specific values an inferring the other parameters. This can include univariate (a single parameter) or multivariate based profiling [12]. Here, we focus on constructing*

*univariate parameter confidence intervals.”*

3. The mathematical model, Equations 1-4, seem to be standard within the blood flow modelling community, and the different types of boundary conditions are described appropriately in the Methods section. For me, as a generalist reader, what is missing is some kind of simulation of the model, with various BC choices to show the reader what the solution of the model looks like, and it would be even useful to show solutions with different parameter values so that we can begin to appreciate, visually at least, how the choice of parameter impacts the solution of the model.

- **This information is valuable to include for a generalized readership, and we have thus added a new figure (Fig 3) to the results section. These results include seven realizations from the Windkessel and structured tree boundary condition parameters, as well as stiffness variation, across pressure, area, and flow in the three vessels. We have also added information about this new material to the main text in the Results section on page 10:**

*“We provide visualizations of several of these PDE solutions for the two boundary condition types in Fig 3. In general, pressure and area dynamics are nearly identical in shape whereas flow waveforms can vary relatively more. Flow signals in the structured tree model also tend to vary more than in the Windkessel model.”*

4. Part of the challenge I have in reading the paper (and I will not be alone as a generalist reader) is that the first results I see are in Table 1, and Figures 3-4 which summarise some principal components and Sobol indices for those principal components for a set of simulations. Without seeing the simulation data it is impossible for a general reader to understand what the main types of data are, and it is impossible to understand how these Sobol indices relate to questions of parameter estimation, parameter identifiability and experimental design.

**- We agree and, following the previous comment and the present criticism, have added a new Fig 3 with realizations from the training data for each of the model outputs.**

5. Figures 8 and 10 present solution of the model "along the profile likelihood" which I take as being a profile-wise prediction. Can you determine the accuracy of these profile-wise predictions by comparing with a full prediction using the full profile log-likelihood? For the moment it is not obvious to me how to interpret the results in Figure 10, for example, where we see that there are relatively small differences between Designs D1, D2 and D3, except we have larger variation with some parameters compared with others. Can the author be clearer about how these prediction intervals are calculated?

**- We agree that this needs clarification. We are providing profile-wise predictions in the sense that we provide the output signals from our model at the specific parameter values obtained from our univariate profile-likelihood confidence intervals. We show output signals for the entire profile (above or below the confidence bound) in grey, and color code output signals within the confidence threshold in color. Here we aim to show how confidence intervals naturally translate to output signals, with parameter values within the univariate profiles corresponding to output signals closer to the measured data (shown in black). The goal of what are now Figs 9 and 11 are to emphasize that when univariate profile-likelihood confidence intervals suggest non-identifiable parameters, all the output solutions are essentially identical, where as identifiable parameter, by definition, lead to variability in the output space. The goal of these figures is to help readers understand the links between input and output spaces during these analyses. We have tried to make this clearer by editing the text in the Results on page 17:**

“*We now investigate how univariate profile-likelihood confidence intervals translate to signals in output space. As shown in Fig 9, MPA pressure predictions are provided using parameter values obtained along the profile likelihood in Fig 8(a). To obtain these pressure predictions, we pass the inferred parameter vector obtained from each profiled parameter through the PCE surrogate.”*

**To consider full profile-likelihood analyses (such as those presented in doi:** **10.1371/journal.pcbi.1011515), we need to consider modulations in the data attributed to either (a) measurement error (as a function of time) or (b) emulation error. This latter is especially relevant here, as the current manuscript does not include measurement error, but does account for the inherent mismatch between the surrogate and the true simulator. We believe this is an important next step, though outside the current scope of the present manuscript. We have elaborated on what needs to be done next in terms of multivariate and full profile-likelihood in the Limitations section on page 29:**

“*We only consider univariate profile-likelihood calculations, yet more conclusive multivariate profile-likelihood methods should be considered in the future [39]. Future applications of this approach with real data will require additional error structure, including a separation between surrogate error, simulator discrepancy, and measurement error [22, 39]. There is also a need for more robust methods for updating the likelihood function based on new experimental data. This includes more statistically informed methods for scaling multiple entries in the likelihood function. For instance, profile-wise analysis as described by Simpson et al. [39] is a necessary next step with this approach.*”

6. A key theme in the work is to use a surrogate model instead of dealing with the PDE model. There is nothing wrong with this, but after reading (and re-reading) the article, I remain to be convinced that I understand the accuracy of the surrogate model. This should be demonstrated more thoroughly in the document to provide confidence that the surrogate model is accurately capturing the PDE solution.

**- We use the mean square relative error (MSRE) in Fig 4 from a set of test data (i.e., PDE solutions not used to train the surrogate) as our reference for surrogate accuracy. These results show that the median MSRE in the Windkessel model consistently below 5%, suggesting a strong agreement between simulator and surrogate predictions. The structured tree model has a larger error, and tends to perform worse for pressure and area emulation. To make this apparent, we now provide detailed emulation and PCA-reconstruction errors in Supplementary Figs 2 and 3. This again shows that emulation and reconstruction error are smaller for the Windkessel model than the structured tree, and that pressure and area emulation are more difficult for the structured tree model. We have also added time-series relative errors in Figs 4 and 5 of the supplementary material (error is relative to the maximum of the data). This shows that the Windkessel model emulation error relatively small for the five signals considered in our experimental design. Structured tree emulation shows a larger relative error, again with worse performance on pressure and area. We have expanded on these findings in the Discussion section on pages 23-24:**

*“Additional variance and error metrics in the Supplementary Material also support the claim that the structured tree boundary conditions provide a more variable simulator. The relative error between emulator predictions and test data show that pressure and area emulation is more difficult in the structured tree than for flow emulation. In addition, relative errors are much lower for the Windkessel model.”*
